# Genomic Relevance of *FGFR2* on the Prognosis of HCV-Induced Hepatocellular Carcinoma Patients

**DOI:** 10.3390/jcm11113093

**Published:** 2022-05-30

**Authors:** Walizeb Khan, Washaakh Ahmad, Anwar M. Hashem, Shadi Zakai, Shafiul Haque, Muhammad Faraz Arshad Malik, Steve Harakeh, Farhan Haq

**Affiliations:** 1 Department of Biosciences, COMSATS University, Islamabad 44000, Pakistan; wjzebkh@yahoo.com (W.K.); wishiahmad@gmail.com (W.A.); famalik@comsats.edu.pk (M.F.A.M.); 2 Department of Medical Microbiology and Parasitology, Faculty of Medicine, King Abdulaziz University, Jeddah 22252, Saudi Arabia; amhashem@kau.edu.sa (A.M.H.); szakai@kau.edu.sa (S.Z.); 3 Vaccines and Immunotherapy Unit, King Fahd Medical Research Center, King Abdulaziz University, Jeddah 22252, Saudi Arabia; 4 Research and Scientific Studies Unit, College of Nursing and Allied Health Sciences, Jazan University, Jazan 45142, Saudi Arabia; shafiul.haque@hotmail.com; 5 King Fahd Medical Research Center, King Abdulaziz University, Jeddah 22230, Saudi Arabia; 6 Yousef Abdul Latif Jameel Scientific Chair of Prophetic Medicine Application, Faculty of Medicine, King Abdulaziz University, Jeddah 22230, Saudi Arabia

**Keywords:** *FGFR2*, HCV+, HCC, cirrhosis, HCV-induced HCC

## Abstract

The Fibroblast Growth Factor Receptors (FGFRs) are known to regulate cancer metabolism in different tumor types, including hepatocellular carcinoma (HCC). Several risk factors are associated with HCC, of which viral infections (Hepatitis B and C) and cirrhosis are prominent. In Pakistan as well as in highly developed countries like the United States, hepatitis C virus HCV infections are most commonly reported in HCC. Here, we aimed to investigate the clinical relevance of FGFR receptors in HCC and their role in HCV-positive HCC cases. 264 HCC samples along with their clinical information and 96 normal liver samples were collected. qPCR was done to estimate the expression of *FGFR1*, *FGFR2*, *FGFR3* and *FGFR4*. Three independent HCV-induced HCC cohorts (containing 293 HCC samples) were used for validation. According to in vitro results, *FGFR1* was upregulated in HCV+ HCC patients. However, in all three independent cohorts of HCC, significant a down-regulation of *FGFR1* was observed. *FGFR2* overexpression was observed in the in vitro cohort as well as in three independent HCC cohorts. Interestingly, a strong correlation of *FGFR2* expression was observed between cirrhosis and HCV in all four HCC cohorts. Our study suggested that *FGFR2* expression can be used to classify HCC patients based on HCV infection. This *FGFR2*-based classification may lead to new therapeutic strategies against HCV-positive HCC subtypes.

## 1. Introduction

Hepatocellular Carcinoma (HCC) is the third leading cause of cancer-related death. It is ranked as the fifth and ninth most common malignant tumor in men and women, respectively [1,2]. Hepatitis C virus (HCV) was historically one of the leading causes of hepatocellular carcinoma (HCC) and liver-related mortality [3]. It is among the most feared long-term complications for HCC patients [4].

Although risk factors associated with HCC are numerous, viral infections (including Hepatitis B and Hepatitis C), aflatoxin exposure, liver inflammation, and cirrhosis are the major factors associated with HCC [5,6]. HCC showed a high prevalence and mortality in the endemic areas of HBV and HCV infections [7,8,9]. Moreover, patients with HBV and HCV co-infection showed a higher probability of HCC development and mortality as compared to patients with HBV infection alone [10,11]. Therefore, defining the mechanisms by which viruses participate in molecular events in HCC development and progression is paramount for identifying new treatment targets.

FGFRs are a family of receptor tyrosine kinases (RTKs) encoded by four different genes (*FGFR1–4*) [12]. Fibroblast growth factor (FGF)/FGF receptor (FGFR) signaling has been reported to be involved in the progression of many cancers, and there is also increasing evidence for the role of FGF signaling in hepatocarcinogenesis [13]. FGFRs are involved in many critical processes including cell proliferation, embryonic development, and other important cellular processes [9]. *FGFR3* and *FGFR4* are the major FGFRs overexpressed in HCC [14]. Deregulated *FGFR3* variants exhibit specific effects in the malignant progression of HCC cells [15]. FGFR has, reportedly, been involved in the progression of many other cancers as well [16]. Interestingly, a very recent study identified *FGFR2* rs2981582 polymorphism in HCV-positive HCC patients, suggesting a linkage between HCV-induced HCC and *FGFR2* polymorphism [17]. Nevertheless, the association between FGFR receptors and HCV-HBV-positive HCC patients is still largely unknown.

Approximately 80% of HCC patients worldwide have HCV infection [14]. Preliminary reports suggest that only a small percentage of HCC patients undergo screening and that they are usually diagnosed at an advanced stage when most therapeutics strategies are futile [18]. Moreover, due to limited financial and technical resources, no genome-wide studies for the classification of HCC patients are reported.

Thus, in the present study, we aimed to investigate the clinical relevance of FGFR receptors in 264 HCC patients from Pakistan. Since HCV is the most prominent risk factor in the Pakistani HCC cohort as well as in developed countries such as the United States, HCV plays an important role and is responsible for the increase in the HCC incidence in this country [19]. According to a recent study, Hepatitis C virus was the leading cause of hepatocellular carcinoma in Egypt (1054 [84%] of 1251 patients) [20]. Therefore we also evaluated the expression of FGFRs in HCV-positive HCC patients. Additionally, we validated our in vitro cohort’s finding in three independent HCC cohorts available in public repositories.

## 2. Materials and Methods

### 2.1. Sample Collection

A total of 264 diagnosed HCC patients from well-reputed hospitals (Holy Family, PIMS and AFIP) in Pakistan were involved in this study. The project was formally approved by the COMSATS University bioethical committee and the respective hospitals. The bio-specimens, mainly preserved biopsy tissues in Formalin-Fixed Paraffin-Embedded (FFPE) blocks from these patients including controls, were collected along with their respective clinicopathological data (including HBV/HCV infections, vascular invasion, tumor size, etc.) from the concerned histopathological laboratory (Table 1). The samples of 264 patients and 36 pools of normal tissues were collected from hospitals of the Rawalpindi/Islamabad Armed Forces Institute of Pathology (AFIP) and Pakistan Institute of Medical Sciences (PIMS).

### 2.2. RNA Extraction and cDNA Synthesis

RNA was extracted from 264 tumor tissues already preserved in formalin-fixed paraffin-embedded (FFPE) blocks. Sixty independent normal liver tissue specimens were used as controls. Total RNA was extracted using TRIzol Reagent (Invitrogen, Carlsbad, CA, USA) and quantified using a Nanodrop spectrophotometer (NanoPhotometer Pearl, IMPLEN, Munich, Germany) while considering the samples below 2.0 of the 260/280 ratio. cDNA was synthesized using the Fire Script cDNA Synthesis Kit (Solis Biodyne, Tartu, Estonia) as per the manufacturer’s instructions. PCR was performed with β-actin primers to confirm the cDNA synthesis. Amplified products were electrophoresed on 2% agarose gel and stained with ethidium bromide for further use.

### 2.3. Primer Designing

Primers for selected receptors of FGF genes (*FGFR1, FGFR2, FGFR3,* and *FGFR4*) mRNA were designed using Integrated DNA Technology (IDC) software, and their specificity was confirmed with NCBI Primer Blast to avoid non-specific binding.

### 2.4. qRT-PCR

Quantitative Real-Time PCR (qRT-PCR) was performed using VeriQuest SYBR Green qPCR Master Mix (Thermo Fisher Scientific, CA, United States). The expression of the target gene was normalized using β-actin as an internal control. The reaction condition included an initial denaturation at 95 °C for 8 min, followed by 35 cycles of denaturation at 95 °C for 30 s and annealing at 58 °C for *FGFR1, FGFR2, FGFR3,* and *FGFR4* in each cycle. The relative expression and fold change were evaluated using the 2^−ΔΔCt^ method.

### 2.5. Data Extraction and Processing

To validate the finding of the in vitro analysis, HCV-positive HCC patients were downloaded from the Geo database, including GSE14323, GSE78737, and GSE6764. These datasets were selected based on their study related to HCV-induced HCC. Geo datasets also contains clinical information including disease state, cirrhosis, viral infection, gender and age (Table S1).

### 2.6. Statistical Analysis

Statistical Analysis was performed using IBM SPSS 21 software (IBM Inc., Armonk, NY, USA). A Wilcoxon Signed Rank test was performed to evaluate the difference between tumor and control. A Mann Whitney U-test and Kruskal Wallis tests were applied. *p*-values < 0.05 were considered statistically significant. Graphpad Prism 5 was used to draw out the graphical representation of the data.

## 3. Results

### 3.1. Expression Analysis of FGFR Genes in In Vitro

The expression of FGFR genes was estimated in a pool of 24 normal samples against 264 HCC-positive samples. The mean age of the patients in the in vitro cohort was 55 years, ranging from 10 to 90 years. Out of 264 HCC patients, 53% were above 55 years of age at the time of diagnosis. Around 39% of the cohort represented poorly differentiated HCC cases, and 50% represented advanced HCC cases. The distribution of these details along with clinical information is presented in Table 1. The clinicopathological analyses were performed for *FGFR1*, *FGFR2*, *FGFR3* and *FGFR4* against age, sex, grade stages, tumor stages, HCV, AFP, cirrhosis, vascular invasion, and cell type (atypical, dysplastic/polygonal, neoplastic) using different statistical approaches.

### 3.2. Association between FGFR Genes and Clinical Features

**Figure 1:**
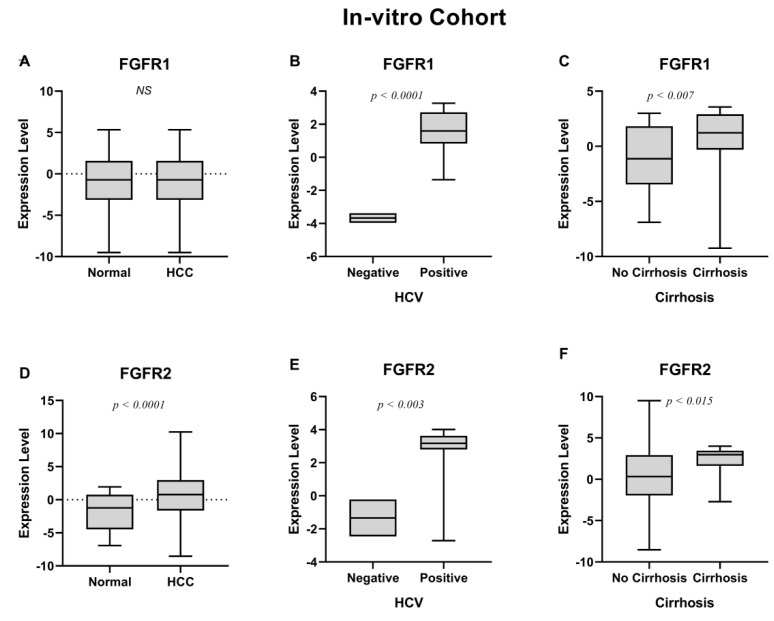
(**A**) *FGFR1* expression in HCC shows no significant *p*-value. (**B**) Upregulation of *FGFR1* in HCV+ patients. (**C**) *FGFR1* upregulation in cirrhosis. (**D**) Overexpression of *FGFR2* in HCC. (**E**) Upregulation of *FGFR2* in HCV+ patients. (**F**) *FGFR2* upregulation in cirrhosis.

**Figure 2:**
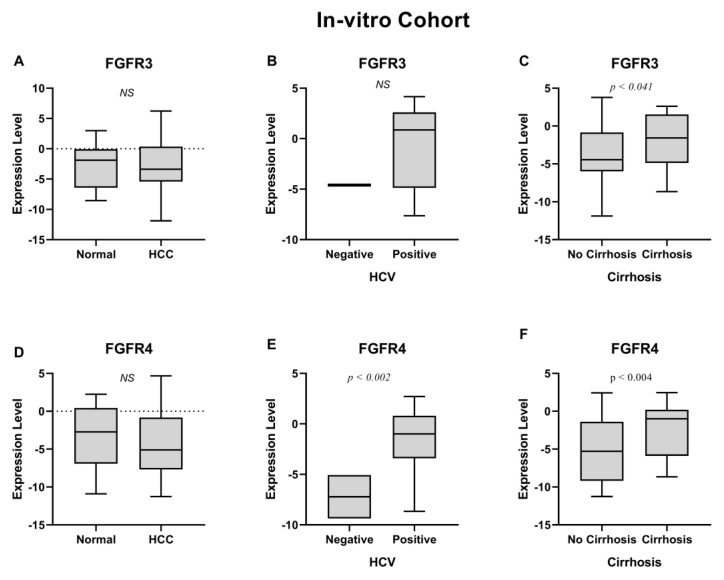
(**A**) *FGFR3* expression in HCC shows no significant *p*-value. (**B**) Regulation of *FGFR3* in HCV+ patients shows no significant *p*-value. (**C**) *FGFR3* is upregulated in cirrhosis. (**D**) *FGFR4* expression in HCC shows no significant *p*-value. (**E**) *FGFR4* upregulation in HCV+ patients. (**F**) Upregulation of *FGFR4* in cirrhosis.

According to the results in Table 2, out of all four FGFRs, only the *FGFR2* gene was significantly overexpressed between HCC and normal samples (*p* < 0.016) (Figure 1D). Additionally, *FGFR2* showed a strong positive association with HCV (*p* < 0.003) (Figure 1E), cirrhosis (*p* < 0.016) (Figure 1F), and age ≤ 50 (*p* < 0.012) (Figure S1A). *FGFR1* overexpression showed a significant association with HCV (*p* < 0.0001) (Figure 1B) and cirrhosis (*p* < 0.0001) (Figure 1C). Moreover, the expression of both *FGFR3* and *FGFR4* was significantly associated with cirrhosis (*p* < 0.05) (Figure 2C,F) while *FGFR4* was also upregulated in HCV-positive HCC patients (*p* < 0.002) (Figure 2E).

The results suggested that all FGFRs were linked to different prognostic characteristics of HCC. Of note, *FGFR1*, *FGFR2* and *FGFR4* upregulation was also significant in HCV-positive HCC patients, suggesting a linkage between the expression of these genes and HCV positivity. Next, the data was collected from the Pakistani HCC cohort; we validated our in vitro findings using an independent dataset of HCV-positive HCC patients using publicly available datasets, to further establish the linkage between FGFRs and HCV infection.

### 3.3. Expression Analysis of FGFR Genes in Validation Cohorts

**Figure 3:**
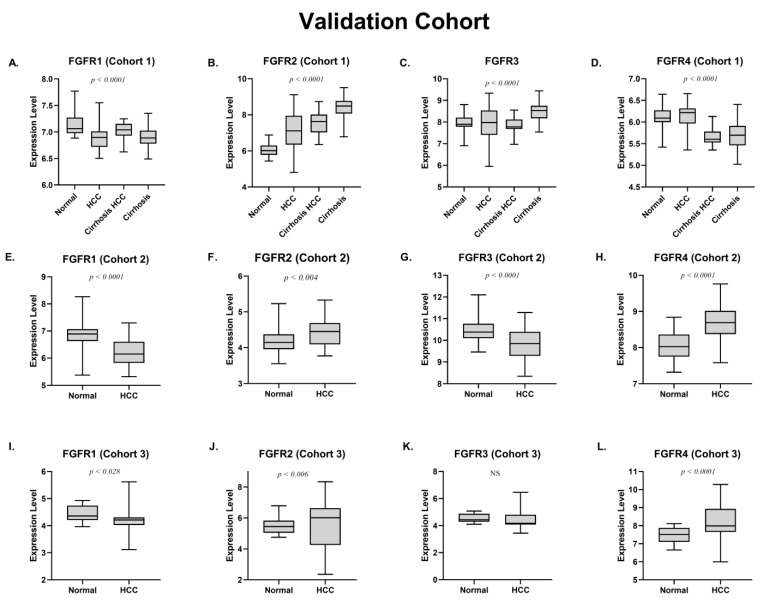
(**A**) Expression analysis of *FGFR1* in different states of disease in cohort 1. (**B**) Expression analysis of *FGFR2* in different states of disease in cohort 1. (**C**) Expression analysis of *FGFR3* in different states of disease in cohort 1. (**D**) Expression analysis of *FGFR4* in different states of disease in cohort 1. (**E**) Expression analysis of *FGFR1* in HCC in cohort 2. (**F**) Expression analysis of *FGFR2* in HCC in cohort 2. (**G**) Expression analysis of *FGFR3* in HCC in cohort 2. (**H**) Expression analysis of *FGFR4* in HCC in cohort 2. (**I**) Expression analysis of *FGFR1* in HCC in cohort 3. (**J**) Expression analysis of *FGFR2* in HCC in cohort 3. (**K**) Expression analysis of *FGFR3* in HCC in cohort 3. (**L**) Expression analysis of *FGFR2* in HCC in cohort 3.

The expression analysis of *FGFR1*, *FGFR2*, *FGFR3*, and *FGFR4* was then validated in three independent validation cohorts consisting of a total of 293 HCC cases (GSE14323, GSE78737, and GSE6764) (Table 3). All three validation cohorts contain viral-induced HCC HCV+ cases, which were also considered as a parameter for the analysis. The validation cohort 1 (GSE14323) consisted of 115 cases including normal samples, patients with cirrhosis and HCC patients with cirrhosis. All HCC patients were also HCV-positive. Consistent with our in vitro findings, *FGFR2* was significantly upregulated in HCV+ HCC cases compared to normal samples (*p* < 0.0001) (Figure 3B). A strong correlation of *FGFR2* expression was also observed in patients with cirrhosis (*p* < 0.0001) (Figure 3B). However, *FGFR1* was significantly downregulated in HCV+ HCC cases (*p* < 0.0001) (Figure 3A). Interestingly, *FGFR4* overexpression was more common in HCC patients with no cirrhosis (Figure 3D).

The validation cohort 2 (GSE78737) consists of 103 cases including normal samples, HCC patients, age, and gender. All HCC patients were also HCV-positive (Table 3). Similarly, a consistent pattern of *FGFR1* and *FGFR2* was observed against HCV. According to the results, *FGFR2* (*p* < 0.004) (Figure 3F) and *FGFR4* (*p* < 0.0001) (Figure 3H) were upregulated, while *FGFR1* (*p* < 0.0001) (Figure 3E) and *FGFR3* (*p* < 0.0001) (Figure 3G) were downregulated in HCV-induced HCC.

The validation cohort 3 (GSE6764) consisted of 75 cases including normal samples, patients with cirrhosis, dysplastic cell type, and HCC patients with cirrhosis. All patients were also HCV-positive (Table 3). Interestingly, a consistent pattern of *FGFR1* and *FGFR2* was observed in HCV-induced HCC cases, with a downregulation in *FGFR1* (*p* < 0.028) (Figure 3I) and an upregulation of *FGFR2* (*p* < 0.006) (Figure 3J) and cirrhosis (*p* < 0.02) (Figure S1B). Moreover, *FGFR3* and *FGFR4* showed a significant upregulation in advanced stages of HCC patients (Figure S1C,D).The results from three independent cohorts suggested a strong positive correlation between *FGFR2* expression and HCV-positive cirrhosis patients as well as HCV-positive HCC patients. Moreover, *FGFR1* expression was downregulated in HCC patients in three independent cohorts.

## 4. Discussion

The present study investigated the clinicopathological characteristics and expression of FGFRs in HCV-induced HCC patients. The results suggested that the upregulation of *FGFR2* was the most common factor among HCV-positive HCC cohorts. Interestingly, a downregulation of *FGFR1* expression was observed in HCC, suggesting different prognostic roles of *FGFR1* compared to other FGFR receptors (i.e., *FGFR2*, *FGFR3* and *FGFR4*). In brief, the expression of *FGFR1* and *FGFR2* may be used as important biomarkers for the subtyping of HCC.

Previous studies suggested that FGFR-mediated signaling played different roles in the cellular mechanisms of HCC [21]. *FGFR1* expression has been reported in multiple cancer types including HCC. However, the differential role of *FGFR1* expression has been observed in cancers. For instance, *FGFR1* is recurrently upregulated in breast cancer, small cell lung cancer, pancreatic cancer, bronchoalveolar cancer, dysembryoplastic neuroepithelial tumor, and prostate cancer [16]. In contrast, some studies showed that *FGFR1* expression was linked to less aggressive types of cancers [22]. Of note, in our previous studies we observed that the overexpression of *FGFR1* in pancreatic cancer was linked to a low-grade tumor and the better survival of patients, suggesting that it may serve as a good prognostic marker [23]. Interestingly, we also observed the downregulation of *FGFR1* in our in vitro cohort of 264 Pakistani HCC patients. Furthermore, *FGFR1* downregulation was also validated in three independent HCC cohorts containing 293 HCC patients. Therefore, we suggested that *FGFR1* expression may represent a distinct subtype of HCC, leading to new therapeutic options.

*FGFR4* is considered an important marker in the proliferation and survival of HCC patients [24]. Previously, *FGFR4* dysregulation was reported in patients with nonalcoholic steatohepatitis or cirrhosis [25,26]. Our study also showed that the overexpression of *FGFR4* was a poor prognostic factor in HCC patients. Similarly, *FGFR2* overexpression was also found in advanced clinical stages and linked with HCC metastasis [27].

The incidence and mortality are increasing worldwide due to the lack of treatment options and late-stage diagnosis [28].

In our study, an overexpression of *FGFR2* was noted in HCV+ cirrhosis patients. Chronic hepatitis C virus (HCV) infects approximately 71 million people worldwide and, beyond the liver damage, is considered to be a systemic disease [29]. In fact, the virus induces chronic liver damage, which can lead to the development of fibrosis, cirrhosis and its complications [30]. Previous studies demonstrated that chronic hepatitis C (HCV) infections caused liver damage, ultimately leading to liver cirrhosis and hepatocellular carcinoma (HCC) [9]. Interestingly, a recent study showed that the rs2981582 variant of *FGFR2* was linked to hepato-carcinogenesis in patients with chronic HCV [17], suggesting that it may act as a potential biomarker for HCV-induced cirrhosis patients.

In Pakistan, due to immense challenges in HCC screening and care, no genomic studies have tried to classify HCC based on major HCC biomarkers. Alarmingly, about 80% of HCC patients are diagnosed at a later stage and have poor prognosis features with a minimal chance of survival [31]. This limits the availability of the clinical data as most of the patients are diagnosed and treated at an advanced stage of cancer. A substantial load of HCV infections is reported in Pakistan. Moreover, Pakistan is among the top countries with an HCV prevalence [32]. Of note, more than 60% of HCC cases reported in Pakistan are HCV-positive, further emphasizing the significance of classifying HCC patients based on HCV infections. Our study revealed that *FGFR2* expression may play a critical role in the progression of HCV-induced cirrhosis patients. Preclinical and clinical studies are required in future to establish the therapeutic implications of *FGFR2* expression in HCV-induced HCC patients. The implication of FGFR2 expression in HCC inducing viral infection can be studied against the therapeutic targets of HCC.

## Figures and Tables

**Table 1:** Clinical features of 264 Pakistani HCC patients.

Characteristics	No. of Patients	% Age
**Age-wise distribution of HCC Patients**		
>50	141	53%
≤50	123	47%
**Gender-based distribution of HCC Patients**		
Male	222	84%
Female	39	14%
NA (Not Available)	3	1%
**HCV-based distribution of HCC Patients**		
Positive	30	11.3%
Negative	6	2%
NA (Not Available)	228	86%
**Grade-based distribution of HCC Patients**		
Grade 1-G1	105	39.7%
Grade 2-G2	105	39.7%
Grade 3-G3	27	10%
**Nuclei appearance based on HCC Patients**		
Pleomorphic	144	54.5%
Non-Pleomorphic	60	22.7%
**HePar1-based distribution of HCC Patients**		
Positive	114	43%
Negative	15	5%
**AFP status-based distribution of HCC Patients**		
High AFP status	27	10%
Low AFP status	9	3.4%
**Cirrhosis-based distribution of HCC Patients**		
Cirrhosis-Present	30	11%
No Cirrhosis-Absent	48	18%
**Vascular Invasion status based on HCC Patients**		
Vascular Invasion-Present	15	5%
No Vascular Invasion-Absent	33	12%

**Table 2:** Association of FGFRs’ expression with clinical features.

Features	*FGFR1*		*FGFR2*		*FGFR3*		*FGFR4*	
	Chi-Square/ Z-Score	Sig.	Chi-Square/ Z-Score	Sig.	Chi-Square/ Z-Score	Sig.	Chi-Square/ Z-Score	Sig.
Normal/HCC patients			−2.419	0.016				
Age group	-	-	−2.510	0.012	-	-	-	-
HCV	−3.750	0.000	−2.951	0.003	-	-	−3.066	0.002
Cirrhosis Status	−2.690	0.007	−2.436	0.015	−2.045	0.041	−2.867	0.004
Vascular Invasion	−3.020	0.003	-	-	-	-	-	-

**Table 3:** Association of FGFRs in three independent cohorts.

Datasets	Genes	*FGFR1*		*FGFR2*		*FGFR3*		*FGFR4*	
	Variable	Chi-Square/ Z-Score	Sig.	Chi-Square/ Z-Score	Sig.	Chi-Square/ Z-Score	Sig.	Chi-Square/ Z-Score	Sig.
GSE14323	Normal vs. Disease	−3.735	0.000	−5.968	0.000	-	-	−2.530	0.011
	Normal vs. HCC	−3.792	0.000	−4.198	0.000	-	-	-	-
	Normal vs. Cirrhosis HCC	-	-	−4.944	0.000	-	-	−3.628	0.000
	Normal vs. Cirrhosis	−3.615	0.000	−6.174	0.000	−3.711	0.000	−3.933	0.000
	Disease States	20.365	0.000	63.154	0.000	27.027	0.000	44.686	0.000
GSE78737	Age	-	-	-	-	-	-	−3.351	0.001
	Normal vs. HCC	−6.056	0.000	−2.901	0.004	−4.018	0.000	−5.630	0.000
GSE6764	Normal vs. HCC	−2.198	0.028	−2.743	0.006	-	-	−2.390	0.0091
	HCC Early/Advance	-	-	-	-	−2.376	0.017	−2.541	0.011
	Normal vs. Cirrhotic Liver	-	-	−2.326	0.020	-	-	-	-
	Normal vs. Dysplastic Liver Tissue	-	-	-	-	−3.314	0.001	-	-
	Disease States	27.046	0.001	18.074	0.021	24.660	0.002	34.390	0.000

## Data Availability

Multiple publicly available datasets are used in the study. The details are provided in the Section 2 where necessary.

## Supplementary Materials

The following supporting information can be downloaded at: https://www.mdpi.com/article/10.3390/jcm11113093/s1, Figure S1: (A) Overexpression of FGFR2 in Age group below 50 in vitro. (B) Overexpression of FGFR2 in Cirrhosis in cohort 3. (C) Overexpression of FGFR3 in Advance stage of HCC in cohort 3. (D) Overexpression of FGFR4 in Advance stage of HCC in cohort 3; Table S1: Clinical Features of 3 independent datasets.
