# Supplementary material for: Genomic Relevance of FGFR2 on the Prognosis of HCV-Induced Hepatocellular Carcinoma Patients
Source: J Clin Med. 2022 May 30;11(11):3093. doi: 10.3390/jcm11113093 (PMC9181427; doi:10.3390/jcm11113093)
Supplement: Supplementary file 1 [file jcm-11-03093-s001.zip › jcm-1709685-supplementary.pdf]

Table S1. Clinical Features of 3 independent datasets

Characteristics	No
**GSE1432**	
**Disease States**	
Normal	19
HCC	38
Cirrhosis-HCC	17
Cirrhosis	41
**Total**	115
**GSE78737**	
**Age**	
Below 50	36
Above 50	67
**Gender**	
Male	95
Female	8
**Disease States**G3	
Normal	66
Tumor	37
**Total**	103
**GSE6764**	
**Disease States**	
**Normal**	10
**HCC**	35
Very Early HCC	8
Early HCC	10
Advanced HCC	7
Very Advanced HCC	10
**Cirrhotic Liver Tissue**	10
**Cirrhotic Liver Tissue from Patients without HCC**	3
**Dysplastic Liver Tissue**	17
Low-Grade Dysplastic Liver Tissue	10
High-Grade Dysplastic Liver Tissue	7
**Total**	75

Supplementary Figure Legends:

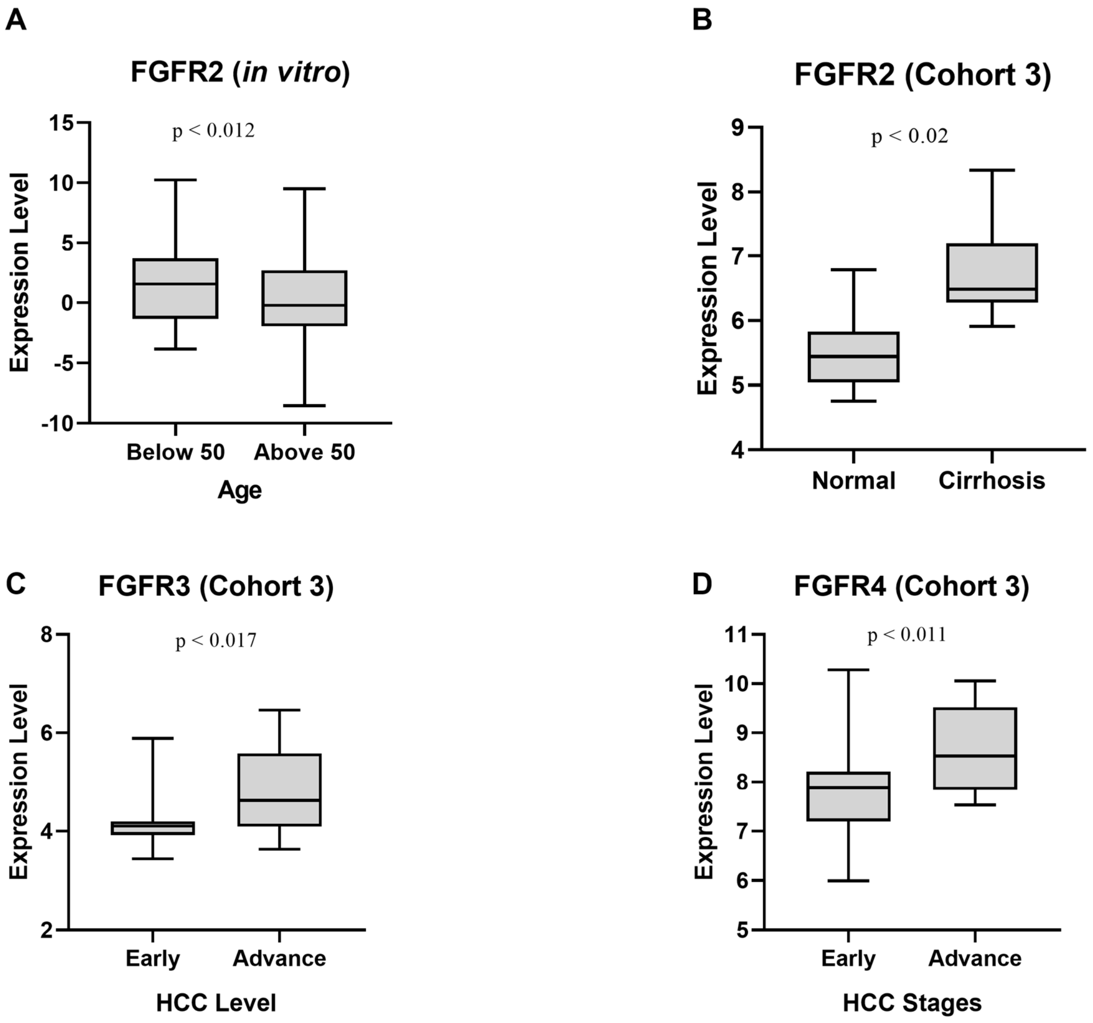

Figure S1. (A) Overexpression of FGFR2 in Age group below 50 *in vitro*. (B) Overexpression of FGFR2 in Cirrhosis in cohort 3. (C) Overexpression of FGFR3 in Advance stage of HCC in cohort 3. (D) Overexpression of FGFR4 in Advance stage of HCC in cohort 3.
